# Eosinophilia and Lung Cancer: Analysis From Real-World Data and Mendelian Randomization Study

**DOI:** 10.3389/fmed.2022.830754

**Published:** 2022-03-09

**Authors:** Zhufeng Wang, Bigui Chen, Yu Fu, Changxing Ou, Qiuping Rong, Xuetao Kong, Wei Xu, Yangqing Deng, Mei Jiang, Jiaxing Xie

**Affiliations:** ^1^National Clinical Research Center for Respiratory Disease, State Key Laboratory of Respiratory Disease, Guangzhou Institute of Respiratory Health, The First Affiliated Hospital of Guangzhou Medical University, Guangzhou, China; ^2^Department of Respiratory and Critical Care Medicine, Yangjiang People's Hospital, Yangjiang, China; ^3^Department of Allergy and Clinical Immunology, National Clinical Research Center for Respiratory Disease, State Key Laboratory of Respiratory Disease, Guangzhou Institute of Respiratory Health, The First Affiliated Hospital of Guangzhou Medical University, Guangzhou, China; ^4^Pulmonary and Critical Care Medicine, Guangzhou Institute of Respiratory Health, National Clinical Research Center for Respiratory Disease, National Center for Respiratory Medicine, State Key Laboratory of Respiratory Diseases, The First Affiliated Hospital of Guangzhou Medical University, Guangzhou, China; ^5^The Princess Margaret Cancer Centre and University Health Network, University of Toronto, Toronto, ON, Canada

**Keywords:** eosinophilia, lung cancer, epidemiology, Mendelian randomization study, prevention

## Abstract

**Background and Objective:**

Growing evidence added to the results from observational studies of lung cancer patients exhibiting eosinophilia. However, whether eosinophils contributed to tumor immune surveillance or neoplastic evolution was unknown. This study aimed to analyze the causal association between eosinophilia and lung cancer.

**Methods:**

The causal effect of eosinophil count on lung cancer from a genome-wide association study (GWAS) was investigated using the two-sample Mendelian randomization (MR) method. Secondary results according to different histological subtypes of lung cancer were also implemented. Meanwhile, we compared the measured levels of blood eosinophil counts among different subtypes of lung cancer from real-world data.

**Results:**

The median absolute eosinophilic count (unit: 10^9^/L) [median (min, max): Lung adenocarcinoma 0.7 (0.5, 15); Squamous cell lung cancer 0.7 (0.5, 1.3); Small cell lung cancer 0.7 (0.6, 1.3); *p* = 0.96] and the median eosinophil to leukocyte ratio [median (min, max): Lung adenocarcinoma 8.7% (2.1, 42.2%); Squamous cell lung cancer 9.3% (4.1, 17.7%); Small cell lung cancer 8.9% (5.1, 24.1%); *p* = 0.91] were similar among different histological subtypes of lung cancer. MR methods indicated that eosinophilia may provide 28% higher risk for squamous cell lung cancer in East Asian [Weighted median method: odds ratio (OR) = 1.28, 95% CI: 1.04–1.57, *p* = 0.02].

**Conclusion:**

Our study suggested that eosinophilia may be a potential causal risk factor in the progression of squamous cell lung cancer in East Asian.

## Introduction

Eosinophils are a type of white blood cell formed from stem cells in the bone marrow. Although normal numbers of eosinophils in the blood are typically <500/mm^3^, they are of great importance (1). On the one hand, together with T lymphocytes, eosinophils participate in the acquired immunity against bacteria, viruses, and tumors (2, 3). On the other hand, eosinophils can release a variety of toxic particles and inflammatory mediators, causing a series of pathophysiologic responses, which leads to inflammation through cytotoxic effects, u*p*-regulated chemokines, and regulation of vascular permeability (3).

It was considered that a higher eosinophilia level had a role to affect carcinogenesis and tumor progression via affecting innate and adaptive immunity (3–8). Whether this association was causal, however, was unknown. A higher eosinophilia level was implicated in the risk of lung cancer, but the direction and magnitude of the association were uncertain across observational studies.

Mendelian randomization (MR) study using genes as instrumental variables (IVs) to study disease association can effectively solve the limitations in traditional observational studies. According to Mendelian laws of inheritance, the alleles are randomly assigned from parents to offspring, which are unlikely to be affected by confounding factors (9, 10). The most studied type of IVs is single nucleotide polymorphism (SNP). IVs refer to a class of variables that are related to exposure factors but unrelated to other confounding factors and have no direct relationship with outcomes (9). If genotype determines phenotype, the genotype is associated with disease development through phenotype. Genotypes can therefore be used as instrumental variables to infer associations between phenotypes and disease outcomes (10).

Therefore, this study aimed to use the MR method, finding SNPs strongly related to blood eosinophilic counts, in order to evaluate the causal association between eosinophilia and lung cancer. Meanwhile, we compared the measured levels of blood eosinophilia counts among different subtypes of lung cancer.

## Methods

### Identify Patients With Lung Cancer and Eosinophilia

A retrospective chart review was completed by searching the electronic medical record to identify all records of individuals of all ages presenting at Yangjiang People's Hospital, a tertiary hospital in Yangjiang City, Guangdong, China, between June 2018 to February 2021, in the inpatient setting. We selected the maximum measured level of blood eosinophil count for each patient who has multi measurements in order to avoid missing any patients who had a history of increased eosinophil counts. Patients with a peripheral blood eosinophil count of ≥0.5 × 10^9^/L would be included. Patients meeting diagnostic criteria for both lung cancer and eosinophilia were included in the study for the major analysis, which should be without a history of other cancers as well. Potential duplicates data were removed based on a combination of factors, including identification card number, home address, age, and gender.

### Clinical Characteristics

Data pertaining to clinical characteristics, including gender, age at admission (year), hospital departments, clinical indicators, and diagnosis information, were collected following a chart review and were compiled for analysis. The clinical characteristics would be described, and the differences would be compared among patients with different subtypes of lung cancer.

### Match Patients With Eosinophilia but Without Lung Cancer

A cohort of patients with eosinophilia but without lung cancer was matched to patients with lung cancer and eosinophilia by using the propensity score matching method, in order to assemble a cohort of patients with similar basic information such as gender and age at diagnosis.

### Mendelian Randomization

The MR approach was based on three assumptions (9): (1) the IVs were strongly related to eosinophilia; (2) the IVs were independent of many confounding factors; (3) the IVs influenced lung cancer only through their impact on eosinophilia rather than other pathways. The procedure of the screening of SNPs was used to meet the first assumption. As body mass index (BMI) and smoking could be confounders for both eosinophilia and lung cancer, we assessed genetic instruments from the largest available meta-analysis of GWASs for BMI (GIANT, European ancestry) (11) and meta-analysis based on over 30 GWASs for smoking (GSCAN, European ancestry) (12). We excluded SNPs that overlapped between different traits considered in our study to reduce potential pleiotropic effects. The causal association estimates from a total of three analysis methods in a two-sample MR approach, including inverse variance weighted average approach (IVW), MR-Egger regression, and weighted median method would be used. The difference among these three methods would be explained in Supplementary Table 1). Additionally, the leave-one-out sensitivity analysis (13, 14) by sequentially eliminating one single SNP at a time would be used to determine if the results were strongly influenced by one SNP. And because different histological subtypes of lung cancer might cause different effects, the causal relationship between eosinophilia and lung adenocarcinoma and squamous cell lung cancer would be analyzed respectively.

### Genetic Variants Associated With Eosinophilic Count

We collected GWAS summary data from Astle WJ. Dataset (ID: ebi-a-GCST004606; 172,275 individuals of European ancestry) contained 179 single nucleotide polymorphisms (SNPs) related to the eosinophilic count. Nine of them were excluded because they did not meet the criteria of a GWAS threshold of statistical significance (*p* < 5 × 10^−8^) and the linkage disequilibrium (LD) r^2^ was greater than 0.001. And we retrieved GWAS summary data from Chen MH. Dataset (ID: ebi-a-GCST90002299; 86,890 individuals of Eastern Asian ancestry) contained 48 SNPs related to eosinophilic count. Two SNPs were excluded for the same reason. A total of four SNPs (rs175705, rs35914442, rs445, and rs7840212, which were corresponded to genes JDP2, BATF; CHD7; CDK6; CCDC26 respectively) were overlapped, which were excluded to limit confounding effects of racial differences. Eligible SNPs explain 8.77 and 3.75% of the variation in eosinophil counts across European and East Asian individuals. As the F statistic is larger than the value of 10, so the instruments could strongly predict the eosinophil counts (15).

### GWAS Summary Data on Lung Cancer

We collect published GWAS summary data on lung cancer from International Lung Cancer Consortium (ILCCO) [Dataset ID: ieu-a-966 with 11,348 cases and 15,861 controls for lung cancer; Dataset ID: ieu-a-965 with 3,442 cases and 14,894 controls for lung adenocarcinoma; Dataset ID: ieu-a-967 with 3,275 cases and 15,038 controls for squamous cell lung cancer; European ancestry]. For each selected SNP associated with the eosinophilic counts, the information on ILCCO was also retrieved. All GWAS data set mentioned would be put in Supplementary Table 2.

### Statistical Analysis

The clinical characteristics comparisons were performed using a Mann-Whitney test or a Kruskal-Wallis test for continuous variables and a chi-square test for categorical variables. A *p*-value of < 0.05 was considered significant. For propensity score matching, a 1:1 matching protocol without replacement (nearest neighbor matching algorithm) was performed. A caliper width equal to 0.2 of the standard deviation of the logit of propensity score was used. A *p*-value of > 0.05 for a given covariate indicates a relatively small imbalance. Binary logistic regression was used to evaluate the relationship between blood eosinophil counts and lung cancer. In MR analysis, the two-sample MR method would be used to estimate the causal relationship between eosinophilia and lung cancer risk. Results were reported by odds ratios (OR) and their 95% confidence intervals (CIs). The MR-Egger regression would be applied to evaluate the pleiotropic test. The funnel plots were provided indications of where there existed directional horizontal pleiotropy for each outcome as well. Statistical analyses were performed with R version 4.0.5 (http://CRAN.R-project.org, R Foundation, Vienna, Austria). All analyses in MR were performed with the R package “TwoSampleMR” (version 0.5.6) (16). A flowchart of the overall design for the present study could be seen in Supplementary Figure 1.

## Results

### Real-World Data Analysis

#### Clinical Characteristics of Patients With Lung Cancer and Eosinophilia

Of the 131,566 unique patients in the inpatient setting between June 2018 and February 2021, a total of 214 patients were diagnosed with lung cancer and eosinophilia. The percentage was higher in males (0.22, 146/65615; *p* < 0.05). It was more common in patients aged 66 to 91 years (0.35, 126/35804; *p* < 0.05) and in the oncology department (1.57, 82/5239; *p* < 0.05). And 3% of patients with lung cancer had mild, moderate, or severe eosinophilia. We reviewed the medical records of these 214 patients, which showed that only 7 patients had a history of allergy, and no patient was caused by parasitic infection or hematological diseases (Table 1).

**Table 1:** Clinical characteristics of patients with lung cancer and eosinophilia (*n* = 214).

**Characteristics**	Patients with lung cancer and eosinophilia (*n* = 214^*^)	**Total inpatients (*n* = 131,566)**	**Percentage of total inpatients (%)**
**Gender** ^†^			
Female	68	65,951	0.10
Male	146	65,615	0.22
**Age at admission (y)^#^^†^**			
68 (37,91)			
37–65	88	74,727	0.12
66–91	126	35,804	0.35
**Hospital department^†^**			
Oncology	82	5,239	1.57
Surgery	102	49,143	0.21
Intensive Care Unit	2	1,608	0.12
Internal medicine	28	34,294	0.08
**Severity of eosinophilia**			
Mild (0.5–1.5 × 10^9^/L)	198	7,214	2.74
Moderate (1.5–5.0 × 10^9^/L)	14	544	2.57
Severe (≥5.0 × 10^9^/L)	2	77	2.60

† *p < 0.05*.

# *Data is presented as median (min, max)*.

* *seven patients had a history of allergy. 81 cases of lung cancer overall; 100 cases of lung adenocarcinoma; 22 cases of squamous cell lung cancer; 11 cases of small cell lung cancer*.

#### The Differences Among Patients With Different Subtypes of Lung Cancer

**Figure 1:**
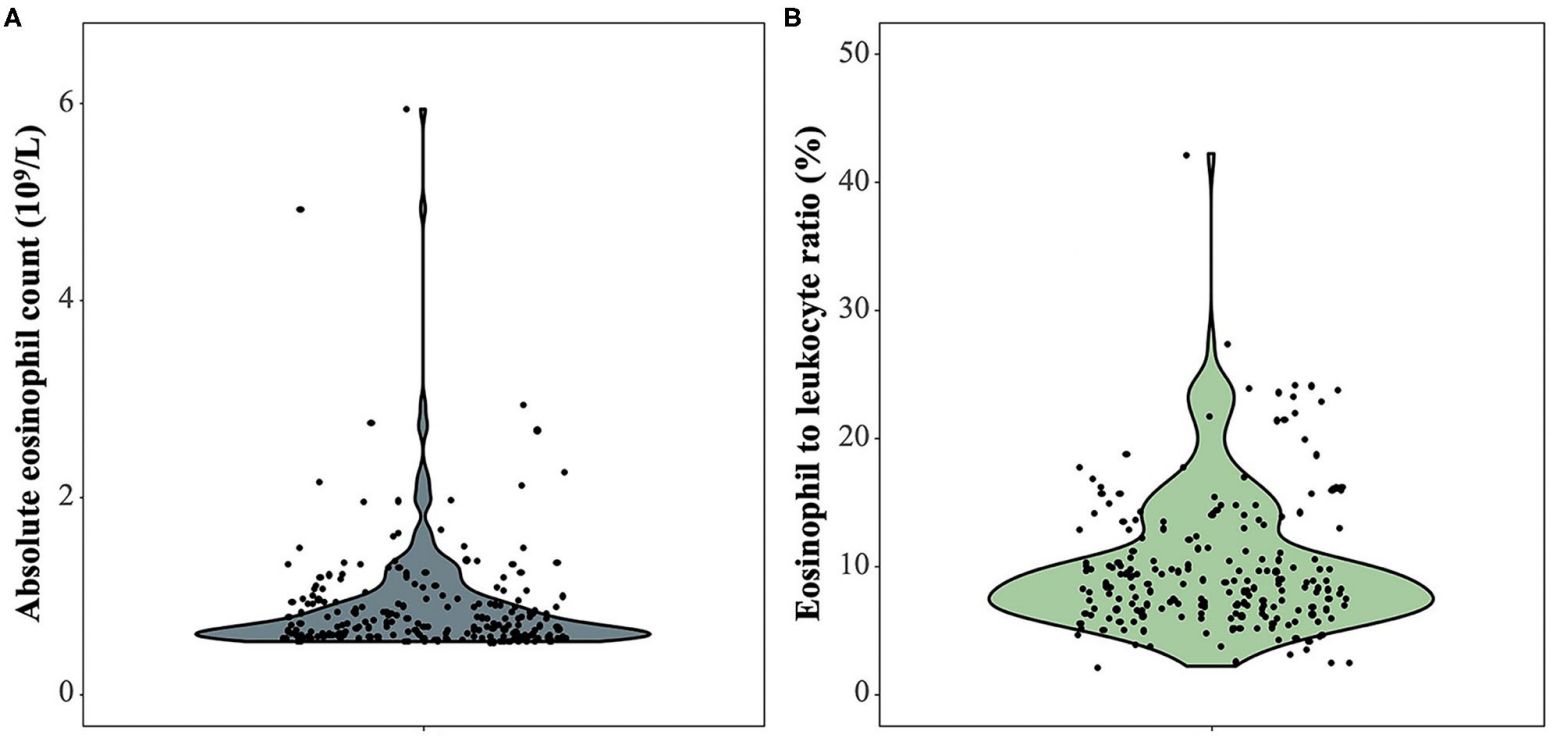
The absolute eosinophilic count and eosinophil to leukocyte ratio in 214 patients with lung cancer and eosinophilia. **(A)** The absolute eosinophilic count, **(B)** Eosinophil to leukocyte ratio. One sample with an absolute eosinophilic count = 15 × 10^9^/L as an outlier is excluded.

**Figure 2:**
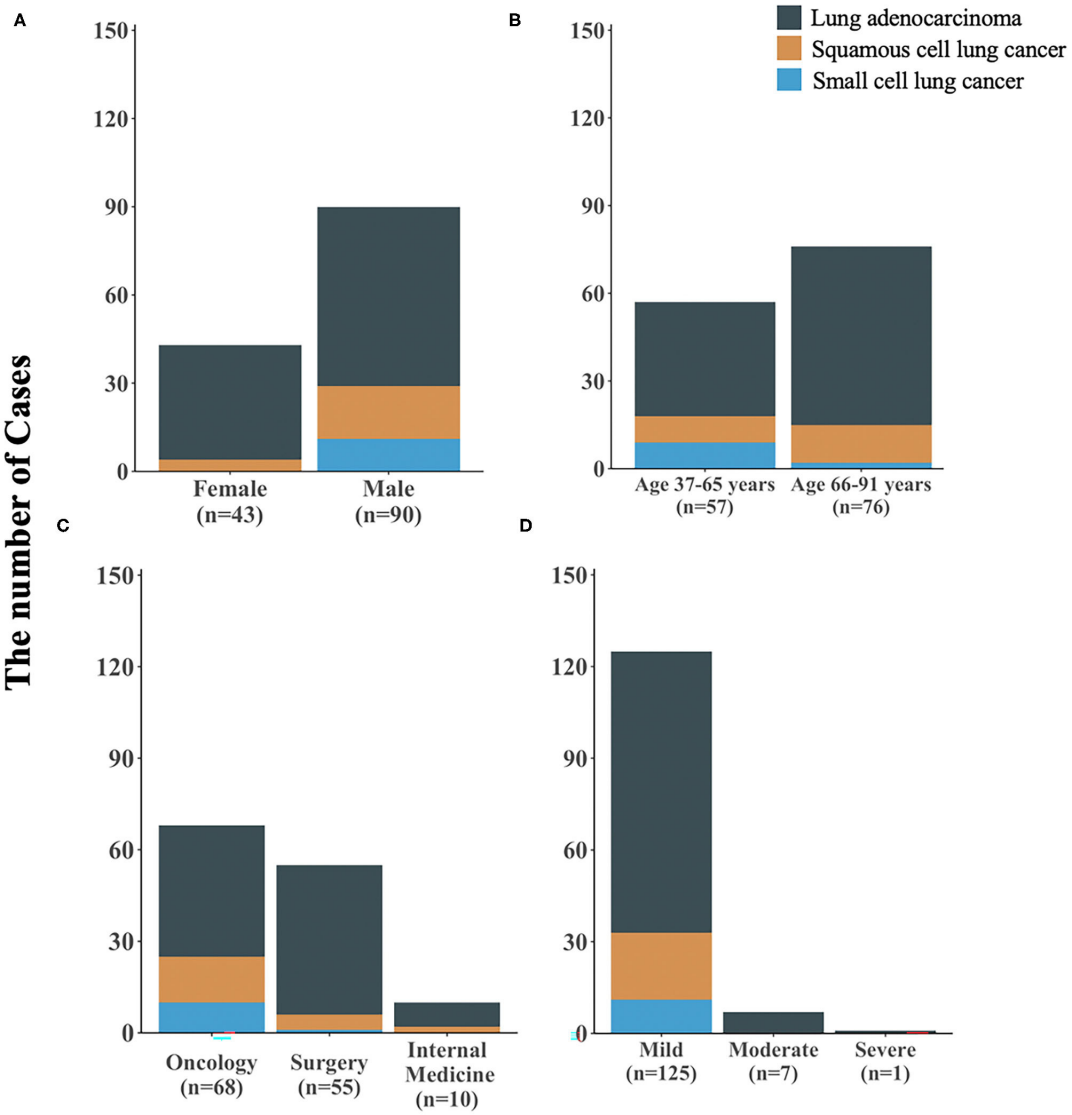
The clinical characteristics of 133 patients with different subtypes of lung cancer and eosinophilia. **(A)** stratified by gender, **(B)** stratified by age, **(C)** stratified by hospital departments, **(D)** stratified by severity of eosinophilia; *n*: sample size.

**Figure 3:**
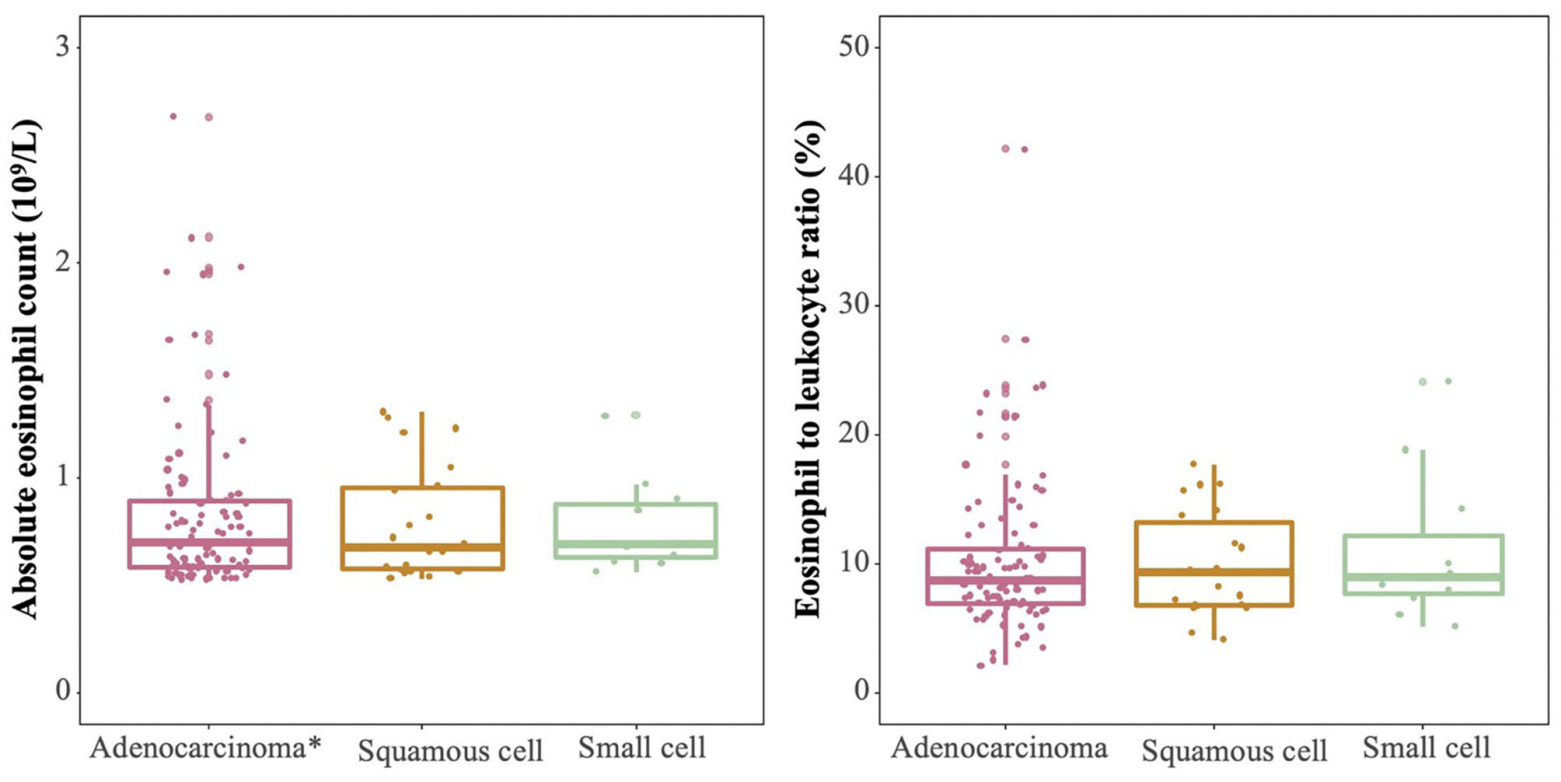
The absolute eosinophilic count and eosinophil to leukocyte ratio in patients with different subtypes of lung cancer and eosinophilia (all *p* > 0.05). *: One sample with an absolute eosinophilic count = 15 × 10^9^/L as an outlier is excluded.

Among 214 patients with lung cancer and eosinophilia, it was found that the median absolute eosinophilic count (unit: ×10^9^/L) was 0.7 (min, max: 0.53, 15), and the median eosinophil to leukocyte ratio was 8.60% (min, max: 2.10, 42.20%) (Figure 1). And 100 cases were diagnosed as lung adenocarcinoma, and 22 cases of squamous cell lung cancer, and 11 cases of small cell lung cancer (Figure 2). The median of the absolute eosinophilic count was similar among different histological subtypes of lung cancer [median (min, max): Lung adenocarcinoma 0.7 (0.5, 15); Squamous cell lung cancer 0.7 (0.5, 1.3); Small cell lung cancer 0.7 (0.6, 1.3); *p* = 0.96]. And the median eosinophil to leukocyte ratio was also close [median (min, max): Lung adenocarcinoma 8.7% (2.1, 42.2%); Squamous cell lung cancer 9.3% (4.1, 17.7%); Small cell lung cancer 8.9% (5.1, 24.1%); *p* = 0.91] (Figure 3).

#### Multivariate Analysis

A total of 214 patients with eosinophilia but without lung cancer was matched by using the propensity score matching method (Supplementary Table 3). Results reported by binary logistic regression showed that the relationship between blood eosinophil counts and lung cancer was not statistically significant [OR: 1.03 (0.85–1.24), *p* = 0.76].

### Mendelian Randomization

At a GWAS threshold of statistical significance, a total of 139 and 34 SNPs were related to eosinophilic counts, represented the European and East Asian populations, and were associated with lung cancer.

**Figure 4:**
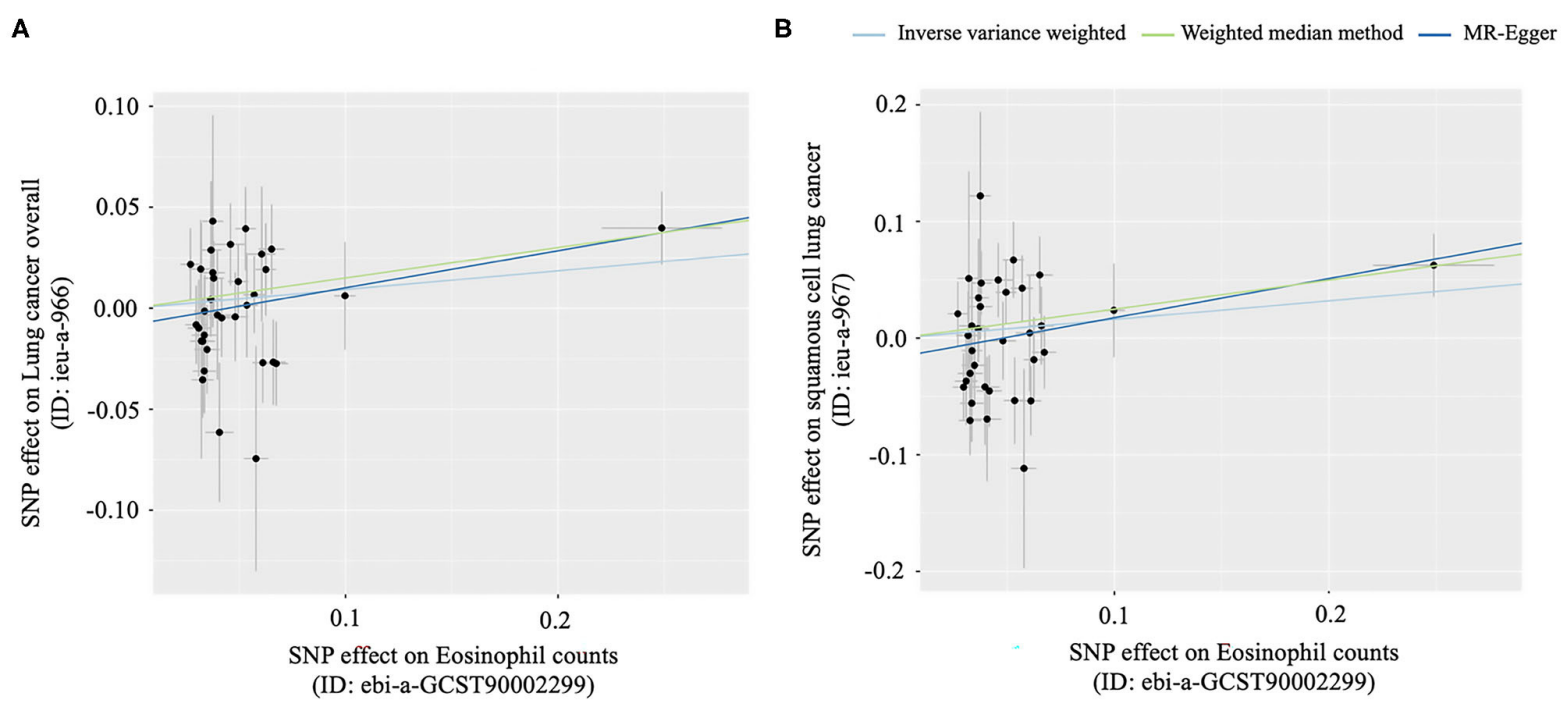
Scatter plots for MR analyses of the causal effect of Eosinophil counts on lung cancer. **(A)** The causal effect of eosinophil counts on lung cancer overall, **(B)** The causal effect of eosinophil counts on squamous cell lung cancer.

It was suggested that a higher eosinophilia level might play a protective role in lung cancer risk in the European population, but it was not statistically significant. However, it might be a risk factor for lung cancer overall in the East Asian population (OR: 1.16, 95% CI: 1.01–1.34, *p* = 0.04), and this association might be mainly seen in squamous cell lung cancer (OR: 1.28, 95% CI: 1.04–1.57, *p* = 0.02), which was statistically significant (Table 2; Figure 4).

**Table 2:** Mendelian randomization estimates of the associations between eosinophilic count and risk of lung cancer overall and histological subtypes.

**Ancestry**	**Outcome**	**IVW method**		**MR-Egger**		**Weighted median method**	
		**OR (95% CI)**	***P*** **value**	**OR (95% CI)**	***P*** **value**	**OR (95% CI)**	***P*** **value**
European (Dataset ID: ebi-a-GCST004606; *n* = 172,275)	Lung cancer overall	0.91 (0.82–1.02)	0.11	0.85 (0.66–1.11)	0.23	1.00 (0.85–1.18)	0.99
	Lung adenocarcinoma	0.91 (0.78–1.05)	0.21	0.80 (0.57–1.13)	0.21	0.87 (0.69–1.10)	0.25
	Squamous cell lung cancer	0.99 (0.84–1.16)	0.87	1.00 (0.68–1.45)	0.98	0.91 (0.72–1.16)	0.44
East Asian (Dataset ID: ebi-a-GCST90002299; *n* = 86,890)	Lung cancer overall	1.10 (0.98–1.22)	0.10	1.20 (1.01–1.42)	0.04^*^	1.16 (1.01–1.34)	0.04^*^
	Lung adenocarcinoma	1.01 (0.86–1.20)	0.87	1.06 (0.81–1.37)	0.69	1.08 (0.87–1.34)	0.50
	Squamous cell lung cancer	1.17 (0.96–1.43)	0.11	1.40 (1.03–1.89)	0.04^*^	1.28 (1.04–1.57)	0.02^*^

* *p < 0.05, statistically significant*.

### Sensitivity Analysis

Among 214 patients with lung cancer and eosinophilia, a total of seven patients had a history of allergy (six patients with dermatitis, and one with asthma). Eosinophilia could be caused by allergy, which might lead to an inaccurate estimate of the relationship between eosinophilia and lung cancer. After restricting to 207 patients who have no history of allergy (77 cases of lung cancer overall; 98 cases of lung adenocarcinoma; 21 cases of squamous cell lung cancer; 11 cases of small cell lung cancer), results were consistent with the main analysis (Supplementary Table 4).

In terms of MR analysis, we illustrated the single causal effect from the SNPs respectively (Supplementary Figures 2, 3). Also, the degree of pleiotropy was small, given that the intercept was close to zero and the *p*-value was >0.05 (Table 3). No directional horizontal pleiotropy was detected for each outcome by the funnel plot as well (Supplementary Figure 4). To reduce potential pleiotropic effects, we excluded SNPs that overlapped between different traits considered in our study and the search of GWAS Catalog as well (Supplementary Table 5). But we found that no SNPs were needed to be excluded. Additionally, none of a single SNP would strongly change the effect of eosinophilia and lung cancer, which was shown by the leave-one-out sensitivity analysis (Supplementary Figures 5, 6).

**Table 3:** Results of the pleiotropy test.

**Population**	**Outcomes**	**MR-Egger method**
		**Intercept**	***P*** **-value**
European	Lung cancer overall	0.003	0.567
	Lung adenocarcinoma	0.005	0.436
	Squamous cell lung cancer	−0.001	0.955
East Asian	Lung cancer overall	−0.008	0.189
	Lung adenocarcinoma	−0.004	0.700
	Squamous cell lung cancer	−0.016	0.149

Considering the potential bias from racial differences, we collected GWAS summary data from Ishigaki K. Dataset (ID: bbj-a-20; 62,076 individuals of East Asian ancestry) contained 20 single nucleotide polymorphisms (SNPs) related to the eosinophilic counts. And we collect published GWAS summary data on lung cancer from Ishigaki K. Dataset [Dataset ID: bbj-a-133 with 4,050 cases and 208,403 controls for lung cancer; East Asian ancestry]. For each selected SNP associated with the eosinophilic counts in East Asian ancestry, the information on Ishigaki K. Dataset was also retrieved. Results from MR suggested that the relationship between increased eosinophilic counts and lung cancer was unclear and it was not statistically significant [IVW: OR = 0.89, 95% CI: 0.55–1.23, *p* = 0.51; MR-Egger: OR = 1.14, 95% CI: 0.20–2.07, *p* = 0.79; Weighted median: OR = 1.04, 95% CI: 0.75–1.35, *p* = 0.75] (Supplementary Table 6).

## Discussion

To the best of our knowledge, this is the first study to investigate the potential causal relationship between eosinophilia and lung cancer based on real-world data and MR study. A potential causal risk effect of eosinophilia on the risk of lung cancer, especially for squamous cell lung cancer, in the East Asian population may exist from the results of our study.

In recent two decades, peripheral blood eosinophilia associated with lung cancer was mainly reported in small cases or case series (16–22). A previous study found that 0.5% of cases of over 2,000 patients with malignancy of all histologic types exhibited eosinophilia (8). Whereas, our results showed that the hospital-based proportion of patients with eosinophilia and lung cancer was 0.2% over the last 3 years.

In 1983, Slungaard et al. (5) first discovered that pulmonary carcinoma cells could produce eosinophilopoietic factors, which were later confirmed to be Interleukin-5 (IL-5). However, the results of the observational study in our study showed that the relationship between lung cancer and eosinophilia was not statically significant. Considering the limitations such as reverse causation, confounding, and measurement error in observational studies, we used the MR method using genes as instrumental variables to study disease association, which may provide more accurate results.

Results of our MR study showed that a higher eosinophilia level may increase the risk of squamous cell lung cancer, especially in the East Asian population, with OR reaching 1.28. When using data from the Japanese population to explore the relationship between eosinophilia and lung cancer, it showed no statistically significant differences. Although results may be influenced by racial differences, it should be paid more attention to the results of the Mendelian randomization study.

A previous study considered that aberrant innate, adaptive, and systemic inflammatory processes could contribute to lung cancer susceptibility (23). Studies also showed that lung cancer risk increased by 1.43 times in patients with pneumonia and 1.76 times in patients with mycobacterium tuberculosis infection (24). Eosinophils played a prominent role in responses to inflammatory, allergic, and immunoregulatory situations. Therefore, the causal risk effect between blood eosinophil count and lung cancer could not be ignored. The association between eosinophilia and lung cancer risk was not significant in the European population. However, studies (24, 25) showed that racial difference could be one of the important factors affecting the risk of lung cancer (24–30). Different histological subtypes of lung cancer could also have different relationships with inflammation (21), which to some extent explained the differences.

Recently Li et al. (31) found that in the presence of allergic airway inflammation, eosinophils promoted the adverse effect of tumor metastasis. This team also found a link between eosinophils and tumor metastasis by knocking out eosinophils in some of the mice. And it was found that the number of tumor metastases was elevated by three to five-fold in mice with inflammation.

In clinical practice, clinicians should raise awareness of blood eosinophil count. Intervention for eosinophilia may prevent the occurrence of lung cancer or paraneoplastic syndrome at the early stage and improve the prognosis to some extent. However, the significance of eosinophilia encountered as an incidental finding in routinely obtained complete blood counts was frequently neglected.

There were some limitations in our study. Firstly, the real-world study was based on one single center. Secondly, due to the few GWAS datasets from East Asian populations, the results of our MR study may be affected by racial differences. Thirdly, the lack of results from GWAS datasets for small cell lung cancer is also a limitation.

Despite the limitations, our study had some strengths. The hospital settings represented in our study could mirror the overall development level of most regions in China to some extent, and even in those developing countries in the world. In addition, this study pointed out that more attention should be paid to the relationship between eosinophilia and lung cancer in clinical practice.

In general, the etiology between eosinophilia and lung cancer is complex, and more biological mechanisms studies are needed.

## Conclusion

We concluded that an increase of eosinophils in peripheral blood, in the absence of other known causes for eosinophilia, such as allergic states, a parasitic infection, or hematological disease, may suggest the presence of a malignant tumor that has metastasized. A potential causal risk effect of eosinophilia on the risk of lung cancer, especially for squamous cell lung cancer, in the East Asian population may exist in our study.

## Data Availability Statement

The original contributions presented in the study are included in the article/Supplementary Material, further inquiries can be directed to the corresponding authors.

## Author Contributions

MJ and JX: conception and design and administrative support. BC and QR: provision of study materials. ZW, BC, YF, CO, QR, XK, WX, and YD: collection and assembly of data. ZW, BC, and YF: data analysis and interpretation. All authors wrote manuscript and approved the final version of manuscript.

## Funding

This work was supported by ZNSA-2020013, ZHONGNANSHAN MEDICAL FOUNDATION OF GUANGDONG PROVINCE and the National Natural Science Foundation of China (82070026).

## Conflict of Interest

The authors declare that the research was conducted in the absence of any commercial or financial relationships that could be construed as a potential conflict of interest.

## Publisher's Note

All claims expressed in this article are solely those of the authors and do not necessarily represent those of their affiliated organizations, or those of the publisher, the editors and the reviewers. Any product that may be evaluated in this article, or claim that may be made by its manufacturer, is not guaranteed or endorsed by the publisher.
