# Supplementary material for: Eosinophilia and Lung Cancer: Analysis From Real-World Data and Mendelian Randomization Study
Source: Front Med (Lausanne). 2022 Mar 9;9:830754. doi: 10.3389/fmed.2022.830754 (PMC8959488; doi:10.3389/fmed.2022.830754)
Supplement: Supplementary file 1 [file Data_Sheet_1.docx]

**Supplementary:**

1. **Supplementary Table 1.** The explanation of three methods in two-sample Mendelian randomization
2. **Supplementary Table 2.** All GWAS data set mentioned
3. **Supplementary Figure 1.** A flowchart of the overall design for the present study
4. **Supplementary Table 3.** Baseline Characteristics after Propensity Score Matching
5. **Supplementary Table 4.** Sensitivity analysis results for restricting to 207 patients who do not have any history of allergy
6. **Supplementary Figure 2.** The single causal effect from the SNPs respectively in the European population
7. **Supplementary Figure 3.** The single causal effect from the SNPs respectively in the East Asian population
8. **Supplementary Figure 4.** Funnel plots for MR analyses of the causal effect of Eosinophil counts on lung cancer
9. **Supplementary Table 5.** The information of the instrumental variables used for Mendelian randomization study
10. **Supplementary Figure 5.** Plots of “leave-one-out” analyses for MR analyses of the causal effect of Eosinophil counts on lung cancer in European
11. **Supplementary Figure 6.** Plots of “leave-one-out” analyses for MR analyses of the causal effect of Eosinophil counts on lung cancer in East Asian
12. **Supplementary Table 6.** Mendelian randomization estimates of the associations between eosinophilic count and risk of lung cancer overall in East Asian population dataset.

**Supplementary Table 1.** The explanation of three methods in two-sample Mendelian randomization

| **Name of methods** | **Explanation** |
| --- | --- |
| Inverse variance weighted | **The conventional inverse variance weighted (IVW) method** calculates a weighted **mean** of the SNP-specific causal association estimates. The IVW method is used when IVs meet the three assumption of MR approach. According to the method given by Burgess et al., the estimate by IVW method could be calculated with ratio estimates and standard errors as follows:  $\hat{\beta}_{IVW}=\frac{\sum_{i = 1}^{M} X_{m}Y_{m}{\sigma_{Y_{m}}}^{-2}}{\sum_{i = 1}^{M} {X_{m}}^{2}{\sigma_{Y_{m}}}^{-2}}$; $se\left( \hat{\beta}_{IVW} \right)= \sqrt{\frac{1}{\sum_{i = 1}^{M} {X_{m}}^{2}{\sigma_{Y_{m}}}^{-2}}}$ |
| Weighted median method | **The weighted median method** calculates a weighted version of the **median** of the SNP-specific causal association estimates. |
| MR-Egger regression | **The MR-Egger regression method** would be applied to evaluate the pleiotropic effects. Because MR-Egger regression allows for horizontal pleiotropic effects. So, it would be more appropriate to use the causal association estimates calculated by the MR-Egger regression method, when IVs do not meet the third assumption of MR approach. |

Supposing genetic variant m (m = 1 … M) was in line with the three assumptions of MR. An average change of X_m_ in the exposure factor was observed with the corresponding variant allele with standard error σ_Xm_ and a Y_m_ logarithmic change in the outcome factor of the corresponding variant allele with standard error σ_Ym_.

**Supplementary Table 2.** All GWAS data set mentioned

| **Trait** | **Dataset ID** | **Author** | **Population** | **Sample size** |
| --- | --- | --- | --- | --- |
| Eosinophil counts | ebi-a-GCST90002299 | Chen MH | East Asian | 86890 |
| Eosinophil counts | ebi-a-GCST004606 | Astle WJ | European | 172275 |
| Eosinophil counts | bbj-a-20 | Ishigaki K | East Asian | 62076 |
| Lung cancer | bbj-a-133 | Ishigaki K | East Asian | 212453 |
| Lung cancer | ieu-a-966 | Wang Y | European | 27209 |
| Squamous cell lung cancer | ieu-a-967 | Wang Y | European | 18313 |
| Lung adenocarcinoma | ieu-a-965 | Wang Y | European | 18336 |
| Body mass index | ieu-b-40 | Yengo, L | European | 681275 |
| Cigarettes smoked per day | ieu-b-142 | Liu M | European | 249752 |

**Supplementary Figure 1.** A flowchart of the overall design for the present study


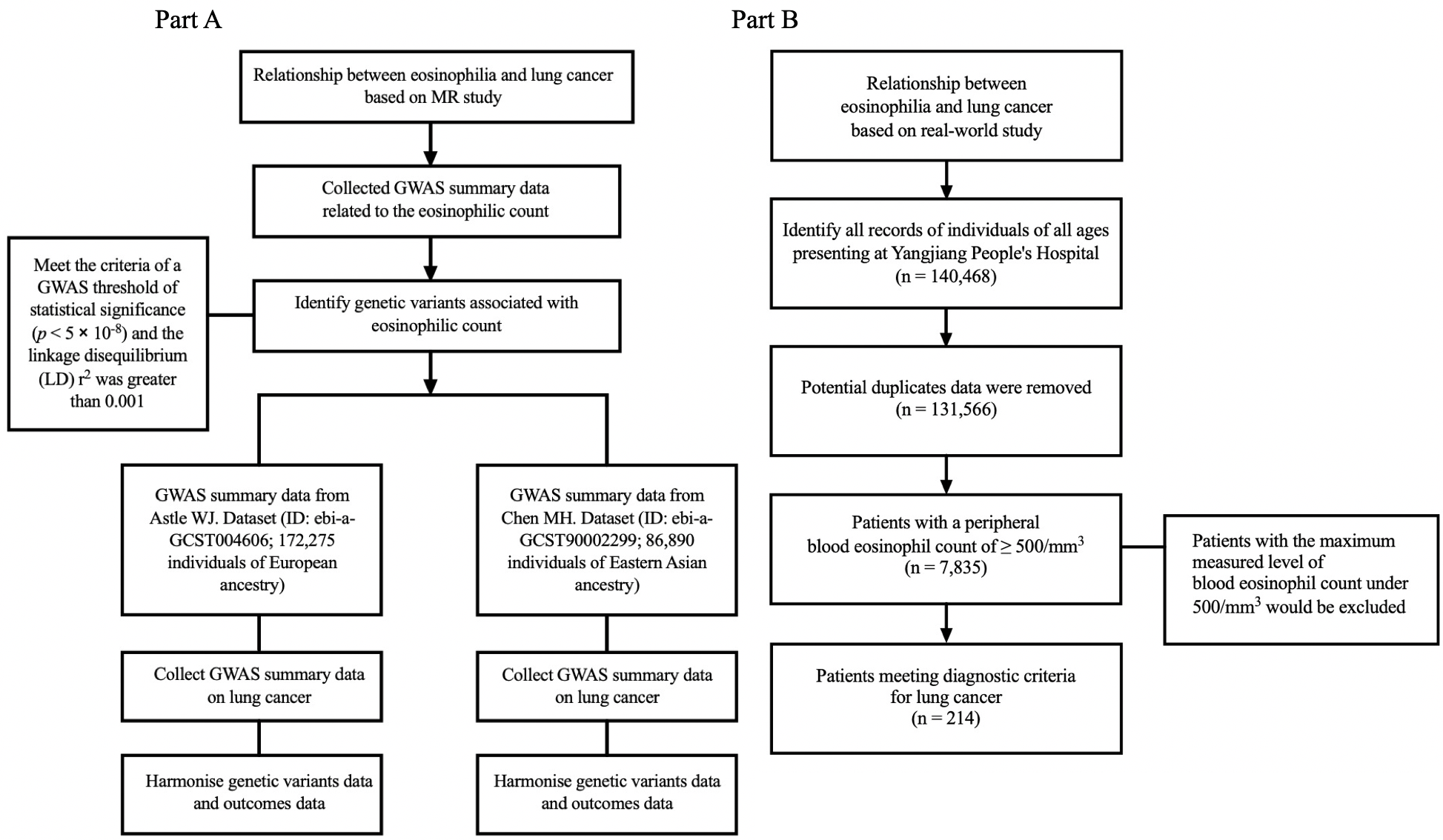


Part A: The study design for evaluating the relationship between eosinophilia and lung cancer based on MR study; Part B: The study design for evaluating the relationship between eosinophilia and lung cancer based on real-world study.

**Supplementary Table 3.** Baseline Characteristics after Propensity Score Matching

| **Characteristics** | **Patients with eosinophilia and lung cancer (n=214)** | **Patients with eosinophilia but without lung cancer (n=214)** | **p-value** |
| --- | --- | --- | --- |
| **Gender** |  |  |  |
| Female | 68 | 68 | 1 |
| Male | 146 | 146 |  |
| **Age at admission (y)#** | 68 (37,91) | 70 (26,93) | 0.14 |
| 37 – 65 | 88 | 88 | 1 |
| 66 – 91 | 126 | 126 |  |
| **Hospital department** |  |  |  |
| Oncology | 82 | 82 | 1 |
| Surgery | 102 | 102 |  |
| Intensive Care Unit | 2 | 2 |  |
| Internal medicine | 28 | 28 |  |
| **Severity of eosinophilia** |  |  |  |
| Mild (0.5 – 1.5 × 10^9^/L) | 198 | 193 | 0.48 |
| Moderate (1.5 – 5.0 × 10^9^/L) | 14 | 20 |  |
| Severe (≥ 5.0 × 10^9^/L ) | 2 | 1 |  |

**Supplementary Table 4.** Sensitivity analysis results for restricting to 207 patients who do not have any history of allergy

|  | Lung adenocarcinoma | Squamous cell lung cancer | Small cell lung cancer | p value |
| --- | --- | --- | --- | --- |
| Absolute eosinophilic count [median (min, max)] (unit: × 10^9^/L) | 0.7 (0.5, 15) | 0.7 (0.5, 1.3) | 0.7 (0.6, 1.3) | 0.87 |
| Eosinophil to leukocyte ratio [median (min, max)] | 8.8% (2.5%, 42.2%) | 9.2% (4.1%, 17.7%) | 8.9% (5.1%, 24.1%) | 0.95 |

**Supplementary Figure 2.** The single causal effect from the SNPs respectively in the European population


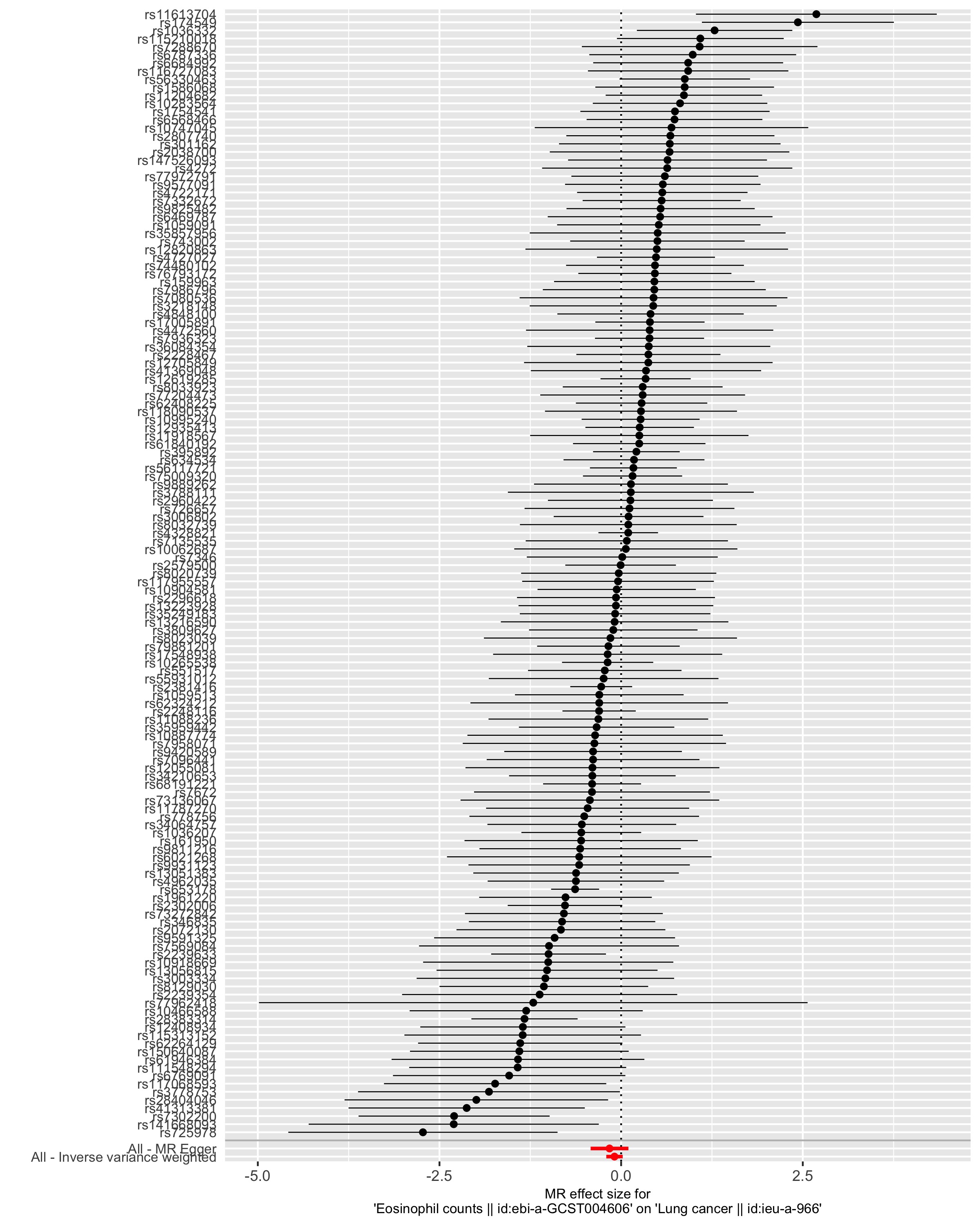


**Supplementary Figure 3.** The single causal effect from the SNPs respectively in the East Asian population


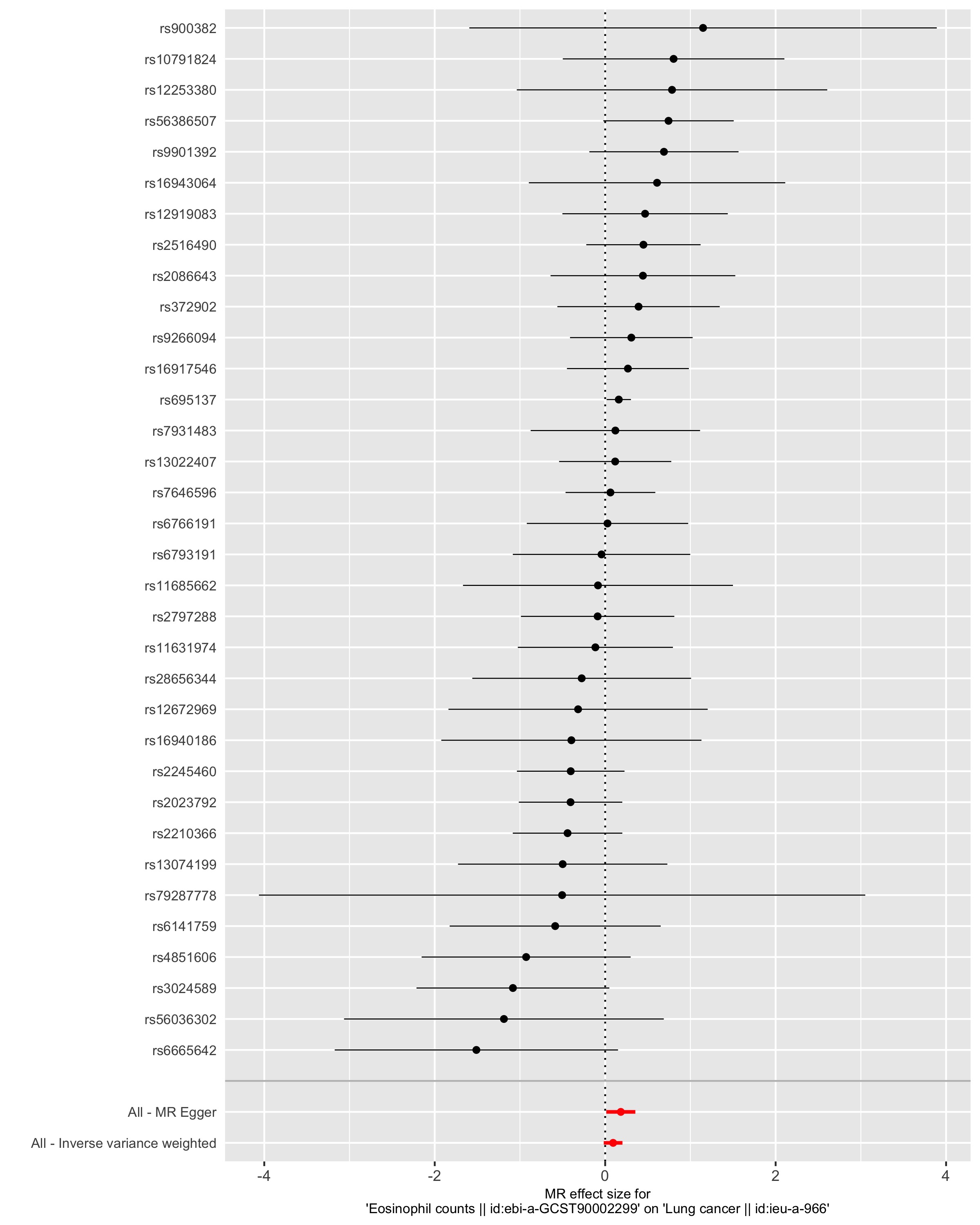


**Supplementary Figure 4.** Funnel plots for MR analyses of the causal effect of Eosinophil counts on lung cancer


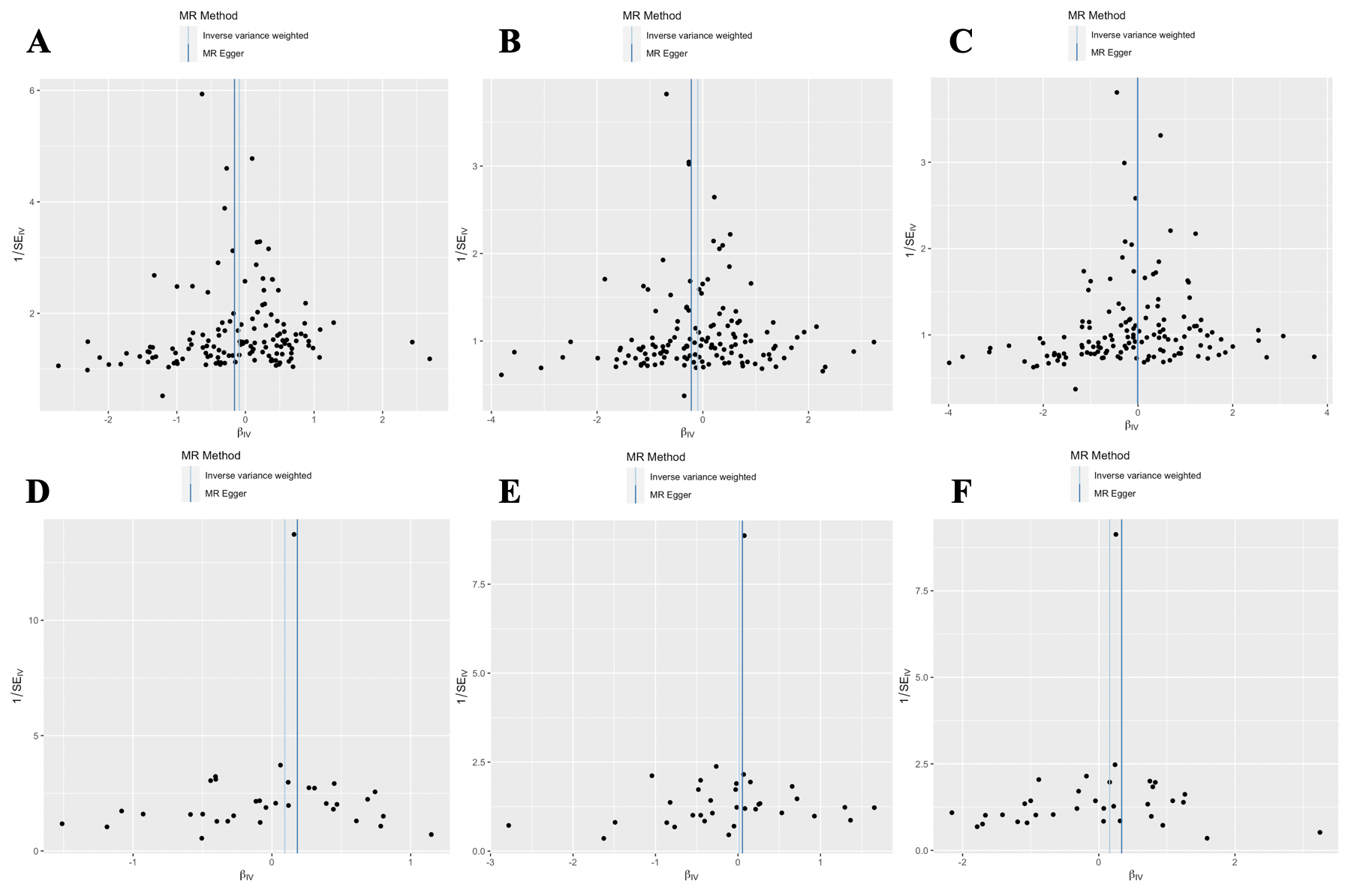


Funnel plots for MR analyses of the causal effect of Eosinophil counts on lung cancer. European: **A,** Lung cancer overall; **B,** Lung adenocarcinoma; **C,** Squamous cell lung cancer; East Asian: **D,** Lung cancer overall; **E,** Lung adenocarcinoma; **F,** Squamous cell lung cancer.

**Supplementary Table 5.** The information of the instrumental variables used for Mendelian randomization study

| Name of SNPs | Location | Cytogenetic region | Mapped gene(s) |
| --- | --- | --- | --- |
| rs6665642 | NA | NA | NA |
| rs11685662 | NA | NA | NA |
| rs56386507 | 2:102354705 | 2q12.1 | IL18R1 |
| rs4851606 | NA | NA | NA |
| rs13022407 | 2:212971482 | 2q34 | MIR4776-1,IKZF2,MIR4776-2 |
| rs2086643 | 2:213037818 | 2q34 | IKZF2 |
| rs13074199 | NA | NA | NA |
| rs900382 | NA | NA | NA |
| rs6766191 | NA | NA | NA |
| rs7646596 | 3:128601502 | 3q21.3 | RPN1,LINC01565 |
| rs6793191 | 3:3100819 | 3p26.2 | IL5RA |
| rs2245460 | 5:132612834 | 5q31.1 | RAD50 |
| rs9266094 | NA | NA | NA |
| rs56036302 | 6:32304379 | 6p21.32 | TSBP1-AS1,TSBP1 |
| rs2516490 | NA | NA | NA |
| rs1126719 | NA | NA | NA |
| rs2210366 | 6:135094070 | 6q23.3 | HBS1L |
| rs2524105 | 6:31265133 | 6p21.33 | HCG27,HLA-C |
| rs6935999 | 6:32424980 | 6p21.32 | TSBP1-AS1,HLA-DRA |
| rs2023792 | 7:20494766 | 7p21.1 | ITGB8,EEF1A1P27 |
| rs12672969 | NA | NA | NA |
| rs13262399 | NA | NA | NA |
| rs28656344 | 8:80392261 | 8q21.13 | RNU6-1213P |
| rs2797288 | 10:9011210 | 10p14 | LINC02676 |
| rs16917546 | 10:62637778 | 10q21.2 | intergenic |
| rs7903779 | NA | NA | NA |
| rs12253380 | 10:8898431 | 10p14 | LINC02676 |
| rs7931483 | 11:76591023 | 11q13.5 | EMSY,LINC02757 |
| rs10791824 | 11:65791795 | 11q13.1 | OVOL1 |
| rs61876250 | 11:325418 | 11p15.5 | IFITM3 |
| rs11631974 | 15:79979658 | 15q25.1 | Metazoa_SRP,BCL2A1 |
| rs16943064 | NA | NA | NA |
| rs3024589 | 16:27349306 | 16p12.1 | IL4R |
| rs16940186 | 16:85976134 | 16q24.1 | LINC02132,LINC01082 |
| rs12919083 | 16:11095073 | 16p13.13 | CLEC16A |
| rs9901392 | 17:4701541 | 17p13.2 | PELP1 |
| rs56305452 | 17:67994384 | 17q24.2 | C17orf58 |
| rs695137 | 17:58699302 | 17q22 | RAD51C |
| rs79287778 | NA | NA | NA |
| rs372902 | 19:39735490 | 19q13.2 | CLC |
| rs6141759 | 20:32589490 | 20q11.21 | NOL4L-DT |
| rs2284029 | NA | NA | NA |
| rs159963 | 1:8444361 | 1p36.23 | RERE |
| rs41313381 | 1:78946283 | 1p31.1 | ADGRL4 |
| rs1036332 | 1:199043349 | 1q32.1 | LINC01221 |
| rs35249183 | 1:12039288 | 1p36.22 | TNFRSF8,RN7SL649P |
| rs3003334 | 1:23873379 | 1p36.11 | CNR2 |
| rs12408934 | 1:64957764 | 1p31.3 | JAK1 |
| rs6684992 | 1:87286317 | 1p22.3 | LINC02801,LMO4 |
| rs11204682 | 1:150623061 | 1q21.3 | ENSA |
| rs111548294 | NA | NA | NA |
| rs3218148 | 1:23525295 | 1p36.12 | E2F2 |
| rs2296618 | 1:198697103 | 1q31.3 | PTPRC |
| rs2786487 | 1:41902668 | 1p34.2 | HIVEP3 |
| rs1754541 | 1:101160327 | 1p21.2 | S1PR1 |
| rs10918669 | NA | NA | NA |
| rs41369048 | 1:220878224 | 1q41 | HLX-AS1 |
| rs1586068 | 2:213033508 | 2q34 | IKZF2 |
| rs12619285 | 2:212959321 | 2q34 | MIR4776-1,IKZF2,MIR4776-2 |
| rs7346 | 2:233204411 | 2q37.1 | INPP5D |
| rs28404046 | NA | NA | NA |
| rs35857956 | NA | NA | NA |
| rs7569084 | 2:65429835 | 2p14 | SPRED2 |
| rs2579500 | 2:96535945 | 2q11.2 | NEURL3,ARID5A |
| rs4848100 | 2:111630955 | 2q13 | ANAPC1 |
| rs346835 | 2:8298563 | 2p25.1 | LINC00299 |
| rs11127153 | 2:28463094 | 2p23.2 | PLB1 |
| rs778756 | 2:61554859 | 2p15 | XPO1,RPS29P10 |
| rs34290285 | 2:241759225 | 2q37.3 | D2HGDH |
| rs34064757 | 3:33005791 | 3p22.3 | GLB1 |
| rs62264129 | 3:112352566 | 3q13.2 | CD200 |
| rs6787336 | 3:3111510 | 3p26.2 | IL5RA |
| rs2960422 | 3:12293492 | 3p25.2 | PPARG |
| rs9825482 | NA | NA | NA |
| rs2228467 | 3:42864624 | 3p22.1 | ACKR2,CYP8B1,KRBOX1 |
| rs11918567 | NA | NA | NA |
| rs115313152 | NA | NA | NA |
| rs73136067 | NA | NA | NA |
| rs6769091 | NA | NA | NA |
| rs116727083 | NA | NA | NA |
| rs4328821 | 3:128597592 | 3q21.3 | RPN1,LINC01565 |
| rs79171715 | NA | NA | NA |
| rs9811216 | 3:169769713 | 3q26.2 | MYNN,ACTRT3 |
| rs7630852 | 3:196781680 | 3q29 | PAK2 |
| rs113473633 | 4:102527974 | 4q24 | NFKB1 |
| rs17005891 | 4:82626709 | 4q21.22 | LINC00575,SCD5 |
| rs68191221 | 4:38675441 | 4p14 | KLF3 |
| rs62324212 | 4:122639784 | 4q27 | IL21-AS1 |
| rs2248116 | 5:132468655 | 5q31.1 | IRF1,IRF1-AS1 |
| rs34908535 | NA | NA | NA |
| rs56330463 | 5:148820448 | 5q32 | HTR4,ADRB2 |
| rs161950 | NA | NA | NA |
| rs1036207 | 5:142119476 | 5q31.3 | NDFIP1 |
| rs1961220 | 5:35844023 | 5p13.2 | IL7R,SPEF2 |
| rs10062687 | 5:10624754 | 5p15.2 | ANKRD33B |
| rs79881201 | 5:111092097 | 5q22.1 | WDR36 |
| rs73272842 | NA | NA | NA |
| rs12055081 | NA | NA | NA |
| rs9266322 | NA | NA | NA |
| rs35959442 | 6:135103041 | 6q23.3 | HBS1L |
| rs3893464 | 6:29967473 | 6p22.1 | HLA-W,MICD |
| rs28383314 | 6:32619436 | 6p21.32 | HLA-DRB1,HLA-DQA1 |
| rs55713716 | 6:376329 | 6p25.3 | DUSP22,IRF4 |
| rs13216590 | NA | NA | NA |
| rs111936749 | NA | NA | NA |
| rs725978 | NA | NA | NA |
| rs62408225 | 6:90246690 | 6q15 | BACH2 |
| rs6568466 | 6:107122949 | 6q21 | BEND3,RNU6-1299P |
| rs10265538 | 7:20502242 | 7p21.1 | ITGB8,EEF1A1P27 |
| rs4722171 | 7:22746098 | 7p15.3 | IL6,MTCYBP42 |
| rs4272 | 7:92607515 | 7q21.2 | CDK6 |
| rs4727027 | 7:149172185 | 7q36.1 | ZNF398 |
| rs13223928 | 7:3131386 | 7p22.2 | CARD11 |
| rs17548938 | 7:50739728 | 7p12.1 | GRB10 |
| rs4639460 | NA | NA | NA |
| rs2302006 | 7:75813412 | 7q11.23 | CCL24 |
| rs3778753 | 7:128939988 | 7q32.1 | IRF5 |
| rs77972791 | 7:37364044 | 7p14.1 | ELMO1 |
| rs150640087 | 7:50376454 | 7p12.2 | IKZF1 |
| rs12705849 | 7:113142501 | 7q31.1 | SMIM30,PPP1R3A |
| rs45577137 | 8:47739071 | 8q11.21 | PRKDC,CEBPD |
| rs12545733 | 8:23099490 | 8p21.3 | TNFRSF10B,TNFRSF10C |
| rs6469787 | 8:118930591 | 8q24.12 | TNFRSF11B |
| rs11787270 | NA | NA | NA |
| rs4472560 | NA | NA | NA |
| rs34173062 | 8:144103704 | 8q24.3 | SHARPIN |
| rs2381416 | 9:6193455 | 9p24.1 | GTF3AP1 |
| rs551517 | 9:110874513 | 9q31.3 | LPAR1 |
| rs147526093 | NA | NA | NA |
| rs55931012 | 9:134084547 | 9q34.2 | WDR5-DT,ARF4P1 |
| rs201272561 | 9:91334470 | 9q22.31 | AUH |
| rs10747045 | NA | NA | NA |
| rs726657 | 9:114934056 | 9q33.1 | DELEC1 |
| rs4962035 | 9:133000647 | 9q34.13 | EEF1A1P5,GFI1B |
| rs10283564 | 9:5075628 | 9p24.1 | JAK2 |
| rs10904581 | NA | NA | NA |
| rs2807740 | 10:28495554 | 10p12.1 | LINC02652 |
| rs10887774 | NA | NA | NA |
| rs7096441 | NA | NA | NA |
| rs61840192 | 10:9001441 | 10p14 | LINC00709,LINC02676 |
| rs9420589 | 10:92690476 | 10q23.33 | HHEX |
| rs3006802 | 10:26447311 | 10p12.1 | APBB1IP |
| rs10995240 | 10:62628871 | 10q21.2 | intergenic |
| rs7080536 | 10:113588287 | 10q25.3 | HABP2 |
| rs1059091 | 11:309127 | 11p15.5 | IFITM2 |
| rs7936323 | 11:76582714 | 11q13.5 | EMSY,LINC02757 |
| rs10466588 | 11:116739533 | 11q23.3 | LINC02702,BUD13 |
| rs10893844 | 11:128315955 | 11q24.3 | ETS1,LINC02098 |
| rs582297 | 11:108294680 | 11q22.3 | ATM |
| rs77204473 | 11:116934348 | 11q23.3 | SIK3 |
| rs174549 | 11:61803910 | 11q12.2 | FADS2,FADS1 |
| rs634534 | 11:65897785 | 11q13.1 | FOSL1 |
| rs12820863 | 12:4209557 | 12p13.32 | HSPA8P5,CCND2-AS1 |
| rs7302200 | 12:56055651 | 12q13.2 | ERBB3,RPS26 |
| rs653178 | 12:111569952 | 12q24.12 | ATXN2 |
| rs1059513 | 12:57095926 | 12q13.3 | STAT6 |
| rs7958071 | NA | NA | NA |
| rs61946384 | NA | NA | NA |
| rs11613704 | 12:871759 | 12p13.33 | WNK1 |
| rs7135535 | 12:92125513 | 12q21.33 | LINC01619 |
| rs9577091 | NA | NA | NA |
| rs9591325 | 13:50237084 | 13q14.2 | DLEU1 |
| rs7986796 | NA | NA | NA |
| rs7332672 | 13:99225217 | 13q32.3 | UBAC2 |
| rs12050099 | NA | NA | NA |
| rs8023039 | NA | NA | NA |
| rs117068593 | 14:92651884 | 14q32.12 | RIN3 |
| rs2239633 | 14:23119848 | 14q11.2 | SLC7A8,CEBPE |
| rs11555542 | 14:93951185 | 14q32.12 | ASB2 |
| rs2038700 | 14:24992783 | 14q12 | STXBP6 |
| rs8020739 | 14:35413286 | 14q13.2 | NFKBIA,DNAJC8P1 |
| rs2010672 | 15:90658042 | 15q26.1 | CRTC3-AS1 |
| rs8033923 | NA | NA | NA |
| rs8032739 | NA | NA | NA |
| rs12935413 | 16:11116590 | 16p13.13 | CLEC16A |
| rs7672 | 16:68260897 | 16q22.1 | PLA2G15 |
| rs3809627 | 16:30091839 | 16p11.2 | TBX6 |
| rs118090537 | 16:88493354 | 16q24.2 | ZFPM1 |
| rs2239354 | 16:57385075 | 16q21 | CCL17,CX3CL1 |
| rs301162 | NA | NA | NA |
| rs7195287 | 16:2786026 | 16p13.3 | PRSS33 |
| rs2072130 | 16:27345077 | 16p12.1 | IL4R |
| rs9931123 | NA | NA | NA |
| rs74480102 | 17:7839283 | 17p13.1 | KDM6B |
| rs111307979 | NA | NA | NA |
| rs34210653 | 17:4632019 | 17p13.2 | ALOX15 |
| rs145947882 | 17:43731839 | 17q21.31 | WHSC1L2P,SOST |
| rs9889262 | 17:49320708 | 17q21.33 | ZNF652 |
| rs115210018 | NA | NA | NA |
| rs113758975 | NA | NA | NA |
| rs17758695 | 18:63253621 | 18q21.33 | BCL2 |
| rs61731111 | 19:3179519 | 19p13.3 | S1PR4 |
| rs76793172 | 19:45856536 | 19q13.32 | SYMPK |
| rs395892 | 19:39735635 | 19q13.2 | CLC |
| rs75009320 | NA | NA | NA |
| rs117955557 | 19:45252110 | 19q13.32 | MARK4 |
| rs141668093 | NA | NA | NA |
| rs36084354 | 19:1079960 | 19p13.3 | ARHGAP45 |
| rs77962418 | 20:31695366 | 20q11.21 | BCL2L1,BCL2L1-AS1 |
| rs6021268 | 20:51524602 | 20q13.2 | NFATC2 |
| rs11088236 | NA | NA | NA |
| rs13051383 | NA | NA | NA |
| rs56117721 | 21:35026289 | 21q22.12 | RUNX1 |
| rs8129030 | 21:35340290 | 21q22.12 | RUNX1 |
| rs3788111 | NA | NA | NA |
| rs13056815 | 22:31272264 | 22q12.2 | LIMK2 |
| rs743002 | 22:41008935 | 22q13.2 | RBX1,Y_RNA |
| rs7288670 | 22:24225858 | 22q11.23 | GGT5 |

**Supplementary Figure 5.** Plots of “leave-one-out” analyses for MR analyses of the causal effect of Eosinophil counts on lung cancer in European


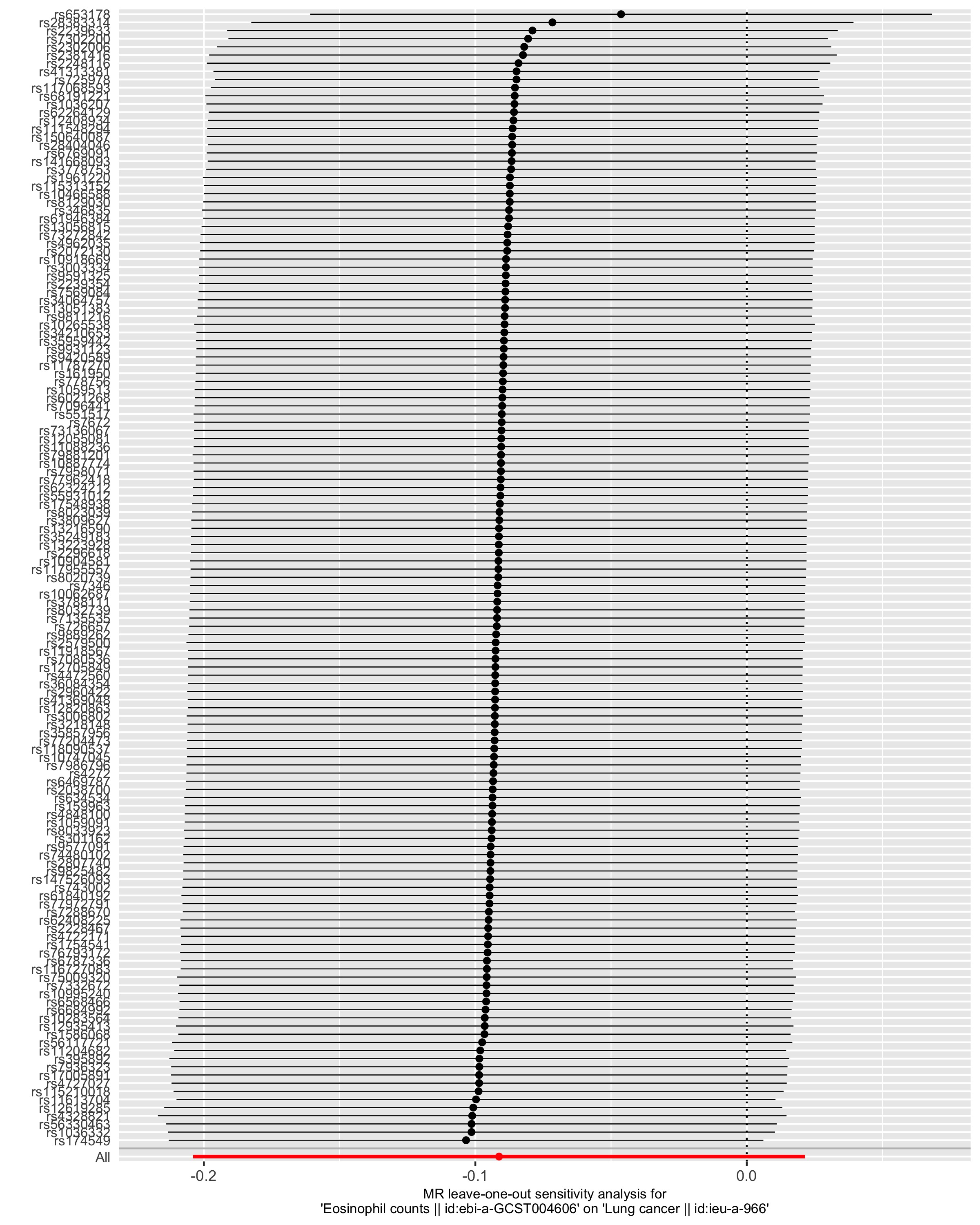


**Supplementary Figure 6.** Plots of “leave-one-out” analyses for MR analyses of the causal effect of Eosinophil counts on lung cancer in East Asian


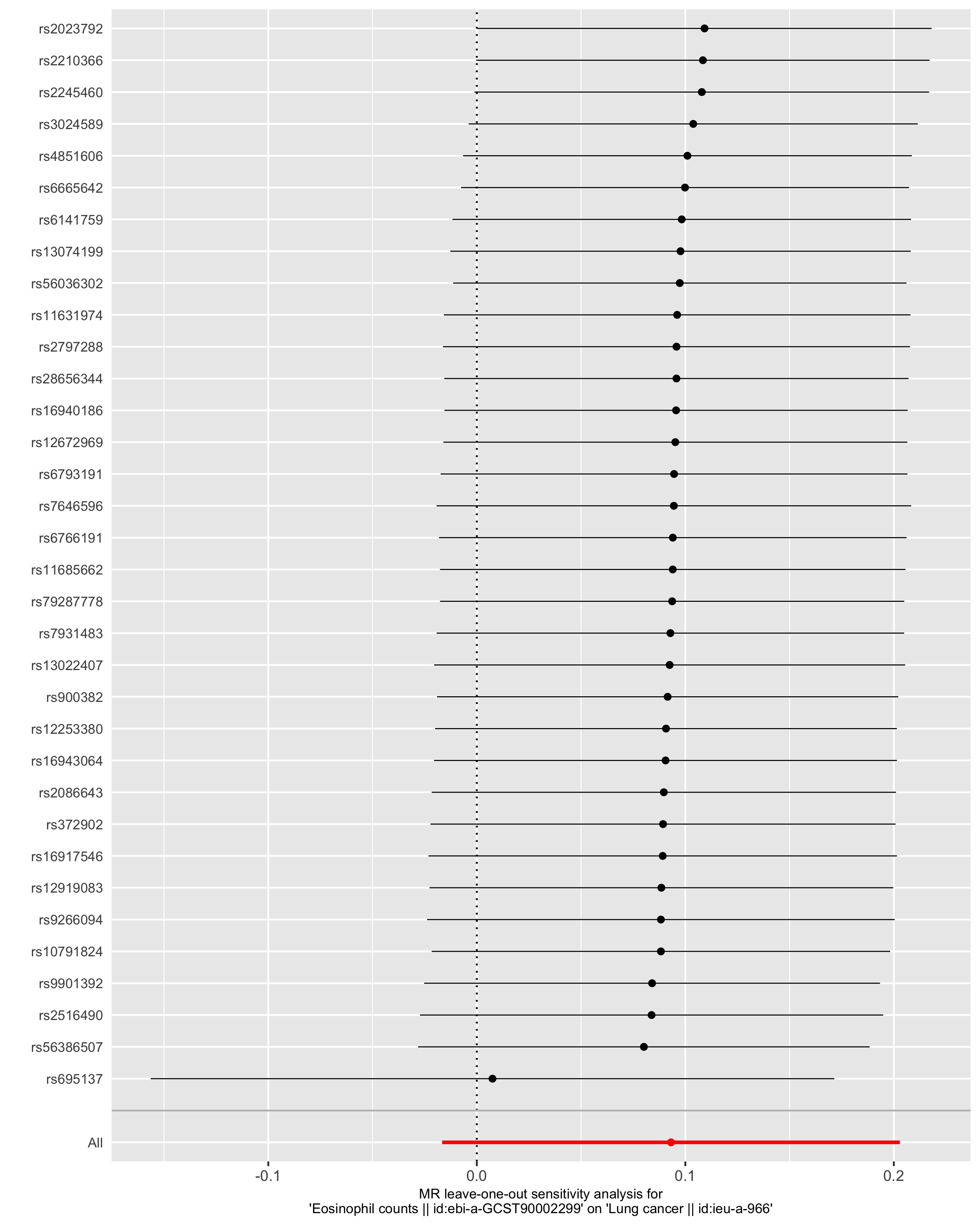


**Supplementary Table 6.** Mendelian randomization estimates of the associations between eosinophilic count and risk of lung cancer overall in East Asian population dataset.

| Ancestry | Outcome | IVW method | | MR-Egger | | Weighted median method | |
| --- | --- | --- | --- | --- | --- | --- | --- |
|  |  | OR (95% CI) | p value | OR (95% CI) | p value | OR (95% CI) | p value |
| East Asian  (Dataset ID: bbj-a-20;  n = 62,076) | Lung cancer overall  (Dataset ID: bbj-a-133;  4050 cases and 208,403 controls for lung cancer) | 0.89 (0.55 – 1.23) | 0.51 | 1.14 (0.20 – 2.07) | 0.79 | 1.04 (0.75 – 1.35) | 0.75 |

*: *p* < 0.05, statistically significant.
